# Extraction of Isoflavones, Alpha-Hydroxy Acids, and Allantoin from Soybean Leaves—Optimization by a Mixture Design of the Experimental Method

**DOI:** 10.3390/molecules28093963

**Published:** 2023-05-08

**Authors:** Sławomir Dresler, Maciej Strzemski, Izabela Baczewska, Mateusz Koselski, Mohammad Bagher Hassanpouraghdam, Dariusz Szczepanek, Ireneusz Sowa, Magdalena Wójciak, Agnieszka Hanaka

**Affiliations:** 1 Department of Analytical Chemistry, Medical University of Lublin, Chodźki 4a, 20-093 Lublin, Poland; maciejstrzemski@umlub.pl (M.S.); i.sowa@umlub.pl (I.S.); magdalenawojciak@umlub.pl (M.W.); 2 Department of Plant Physiology and Biophysics, Institute of Biological Sciences, Maria Curie-Skłodowska University, Akademicka 19, 20-033 Lublin, Polandagnieszka.hanaka@mail.umcs.pl (A.H.); 3 Department of Horticulture, Faculty of Agriculture, University of Maragheh, Maragheh 5518183111, Iran; 4 Chair and Department of Neurosurgery and Paediatric Neurosurgery, Medical University of Lublin, 20-090 Lublin, Poland

**Keywords:** DoE, extraction efficiency, antioxidant, *Glycine max*, phytoestrogens

## Abstract

Soybeans are commonly known as a valuable source of biologically active compounds including isoflavones as well as allantoin and alpha-hydroxy acids. Since these compounds exhibit skin therapeutic effects, they are widely used in the cosmetic and pharmaceutical industries. The presented paper shows the optimization of three solvent systems (ethanol, water, and 1,3-propanediol) to increase the extraction efficiency of isoflavones (daidzin, genistin, 6″-*O*-malonyldaidzin, 6″-*O*-malonylglycitin, 6″-*O*-malonylgenistin), allantoin, and alpha-hydroxy acids (citric acid, malic acid) from soybean leaves. A simplex centroid mixture design for three solvents with interior points was applied for the experimental plan creation. Based on the obtained results of metabolite extraction yield in relation to solvent composition, polynomial regression models were developed. All models were significant, with predicted R-squared values between 0.77 and 0.99, while in all cases the model’s lack of fit was not significant. The optimal mixture composition enabling the maximization of extraction efficiency was as follows: 32.9% ethanol, 53.9% water, and 13.3% propanediol (*v*/*v*/*v*). Such a mixture composition provided the extraction of 99%, 91%, 100%, 92%, 99%, 70%, 92%, and 69% of daidzin, genistin, 6″-*O*-malonyldaidzin, 6″-*O*-malonylglycitin, 6″-*O*-malonylgenistin, allantoin, citric acid, and malic acid, respectively. The solvent mixture composition developed provides a good extraction efficiency of the metabolites from soybean leaves and high antioxidant properties.

## 1. Introduction

Soy (*Glycine max* (L.) Merr., Fabaceae) is an annual plant of great utilitarian importance. The plant provides raw material containing significant amounts of protein, fats, saponins [1], allantoin [2], and isoflavones [1,3]. The presence of these compounds makes soybeans useful in the production of high-protein foods, oil, and pharmaceutical and cosmetic preparations [4]. In the cosmetic industry, extracts containing soy isoflavones (especially aglycones, such as genistein and daidzein) are particularly desirable because of their ability to delay skin aging by inhibiting collagen degradation and increasing the levels of transforming growth factor β (TGF-β). The last process is responsible for the production of an extracellular matrix and stimulates fibroblast proliferation [5,6]. In addition, isoflavones display antioxidant activity. They mitigate the effects of skin exposure to UVB radiation, prevent keratinocyte apoptosis [7], and increase hyaluronic acid synthesis, which benefits the skin water content [8]. Allantoin is also a compound commonly used in cosmetic preparations [9]. It stimulates tissue regeneration through fibroblast proliferation, collagen, and elastin synthesis [10]. The main natural sources of allantoin are plants of the Boraginaceae family [11], including *Symphytum officinale* [12]. However, it has been shown that not only seeds or roots but also aerial parts of soybean plants can be a valuable source of this substance [2,13]. In addition, the biological effect exerted by soybean extracts is due to the presence of primary metabolites (e.g., Krebs acids). It has been shown that alpha-hydroxy acids, such as citric acid and malic acid, can modulate skin keratinization and positively influence the content of elastin, collagen, and glycosaminoglycans. These properties allow these simple plant metabolites to be used as photoprotective substances, positively influencing skin hydration and reducing acne lesions and wrinkles [14,15].

Solvents are crucial factors in the metabolite extraction efficiency in plants [16]. These compounds vary significantly in their solubility properties; thus, the selection of an appropriate solvent mixture brings several difficulties, including proper solvent composition and the proportion chosen [16,17,18]. In this context, the design of experiment (DoE) approach for mixture composition optimization is considered a valuable tool for increasing the extraction efficiency by solvent composition optimization [19,20].

Generally, organic extrahents composed of methanol, acetone, acetonitrile, or dimethyl sulfoxide are suitable for the extraction of most polyphenols, e.g., isoflavones, from plant material [21]. It should be noted that organic mixtures, because of their high human toxicity, are not suitable for direct use in phytotherapeutic (i.e., medical and cosmetic) product preparation, and they are formally listed as prohibited constituents of cosmetics [22]. Moreover, these solvents are not suitable for the extraction of other compounds with therapeutic effects, such as allantoin from soybean. Therefore, the reduction in the usage of toxic organic solvents in favor of new non-toxic solvents has recently attracted much attention. These highly desired safe solvents include alkanediols, such as propanediol, which also exhibit functional skin activity as valuable humectants [17,23,24,25].

Soybean seeds are the main exploited part of the plant subjected to extraction optimization (Table S1), while the leaves left after plant processing are considered waste. However, the rational utilization of by-products is in line with the current trend of “zero waste”, and such by-products may be a rich source of useful biomolecules, e.g., additives for cosmetics [26].

Therefore, the main goal of this paper was to outline the application of the polynomial models developed for the effective extraction of the main active metabolites from soybean leaves, including isoflavones, allantoin, and alpha-hydroxy acids. These compounds differ in terms of polarity, and because of their cosmetic usage, skin- and health-friendly extrahents are greatly wanted. Using the surface response method, the optimization of solvent composition has been performed.

## 2. Results and Discussion

### 2.1. Metabolite Content in Soybean Leaves

Based on the biological activity and skin therapeutic effect, three types of metabolites have been considered in this study—isoflavones, ureide (allantoin), and alpha-hydroxy acids. Quantitative analyses of these compounds were carried out using liquid chromatography as the most recommended method for plant metabolite investigations [27,28,29]. However, due to differences in the polarity of the components, different conditions for separation were required. According to the literature, isoflavones are usually separated using reverse phase systems (the RP C18 column), and the mobile phase is composed of water with acetonitrile or methanol with an addition of acetic or formic acid [30,31]. In turn, highly polar allantoin and alpha-hydroxy acids are analyzed using RP-type beds and water with phosphate buffer as an eluent [32,33] or by means of specific columns including HILIC-type and ion exchange/exclusion fillings [34]. Therefore, in our study, two chromatographic systems were used to investigate the aforementioned analytes.

**Figure 1:**
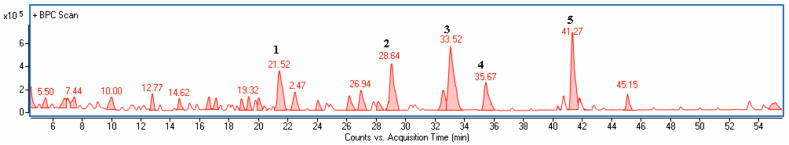
A representative BPC chromatogram of the soybean leaf extract: (**1**) daidzin; (**2**) genistin; (**3**) 6″-*O*-malonyldaidzin; (**4**) 6″-*O*-malonylglycitin; (**5**) 6″-*O*-malonylgenistin.

The ultra-high-performance liquid chromatography with mass spectrometry (UHPLC-MS) analysis showed an isoflavone profile consistent with that reported in the literature for leaf extracts [35]. As can be seen in Figure 1, glucosides (daidzin, genistin) and 6″-*O*-malonyloglucosides (6″-*O*-malonyldaidzin, 6″-*O*-malonylglycitin, 6″-*O*-malonylgenistin) were the dominant isoflavones. Mass data are summarized in Table S2 and Figure S1.

The total amount of isoflavones in plant material was based on exhaustive extraction ranging between 0.18 and 0.72 mg·g^−1^ DW, and the achieved values correspond to previous studies [2,36]. A representative HPLC-DAD chromatogram of alpha-hydroxy acids and allantoin is presented in Figure S2. Total allantoin, malic acid, and citric acid contents reached 5.3, 13.4, and 8.0 mg·g^−1^ DW, respectively. The obtained results of acid content were similar to previous reports [37], while the content of allantoin was almost 3-fold higher in comparison with soybean leaves exposed to Sr [2]. The chemical structures of the analyzed compounds and the results of quantitative analysis are summarized in Table 1.

In comparison, with the accumulation of isoflavones including daidzin (0.38–1.44 mg·g^−1^ DW), genistin (0.37–1.44 mg·g^−1^ DW), 6″-*O*-malonyldaidzin (0.23–0.82 mg·g^−1^ DW), and 6″-*O*-malonylgenistin (0.47–1.42 mg·g^−1^ DW) reported before in soybean seeds [3], it was assumed that leaves of soybean plants could be considered a valuable source of isoflavones.

### 2.2. Polynomial Regression Model Development

To extract metabolites, well-adjusted solvents are necessary. Here, a three-solvent system containing ethanol (EtOH—X_1_), water (H_2_O—X_2_), and propanediol (X_3_) has been tested for soybean leaf extraction optimization (Table 2). Table S3 shows the summary of the model statistics for the isoflavone, allantoin, and alpha-hydroxy acid extraction mixture. The models were optimized by removing insignificant components (*p*-value < 0.05) while keeping the non-fit statistics at an insignificant level. The polynomial equation models developed were highly significant. Except for allantoin (*p*-value of 0.0005), the *p*-values were below 0.0001. The determination coefficients *R*^2^ (above 0.92), as well as predicted *R*^2^ (ranging from 0.78 to 0.99), which were in reasonable agreement with the adjusted *R*^2^ (the difference is less than 0.2), made these models adequate for prediction. Additionally, a high signal-to-noise ratio (Adeq Precision above 4) in all the developed models indicates an adequate signal level and the fact that they could be used to navigate the design space [38].

### 2.3. Solvents’ Effect on Extraction Efficiency

The yield of a single compound extraction process strictly depends on the physical and chemical properties of extrahents. Here, a three-solvent system was used containing EtOH, H_2_O, and propanediol, which present different dipolar moments of 1.69 D, 1.85 D, and 2.52 D, respectively [18,39]. Additionally, the surface tensions (EtOH: 22.3; H_2_O: 71.9; propanediol: 47.8 J·m^−2^) and contact angles (EtOH: 18.8; H_2_O: 85.6; propanediol: 81.3° at 0 ms) (data not published) varied significantly between the solvents. Such a selection of extrahents determined a different penetration ability and wet solid surface [17].

**Figure 2:**
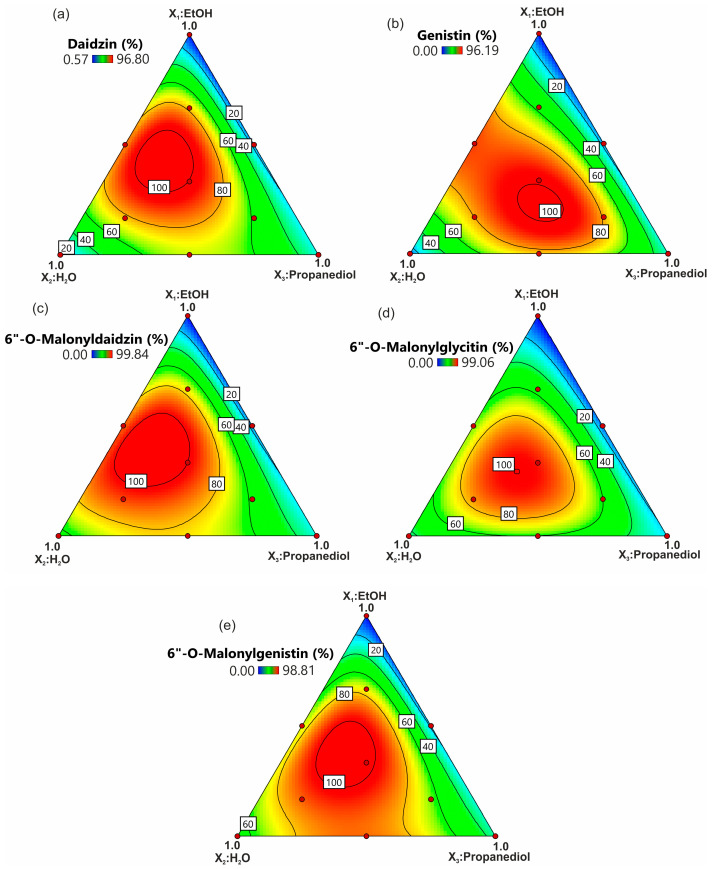
The response contour plot of the impact of the solvent composition on the extraction rate (the percentage of the total amount) of (**a**) daidzin, (**b**) genistin, (**c**) 6″-*O*-malonyldaidzin, (**d**) 6″-*O*-malonylglycitin, and (**e**) 6″-*O*-malonylgenistin.

**Figure 3:**
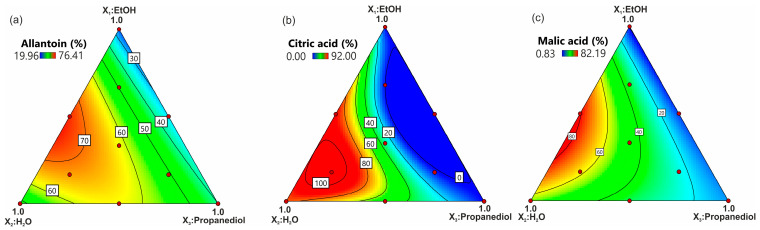
The response contour plot of the impact of the solvent composition on the extraction rate (the percentage of the total amount) of (**a**) allantoin, (**b**) citric acid, and (**c**) malic acid.

**Figure 4:**
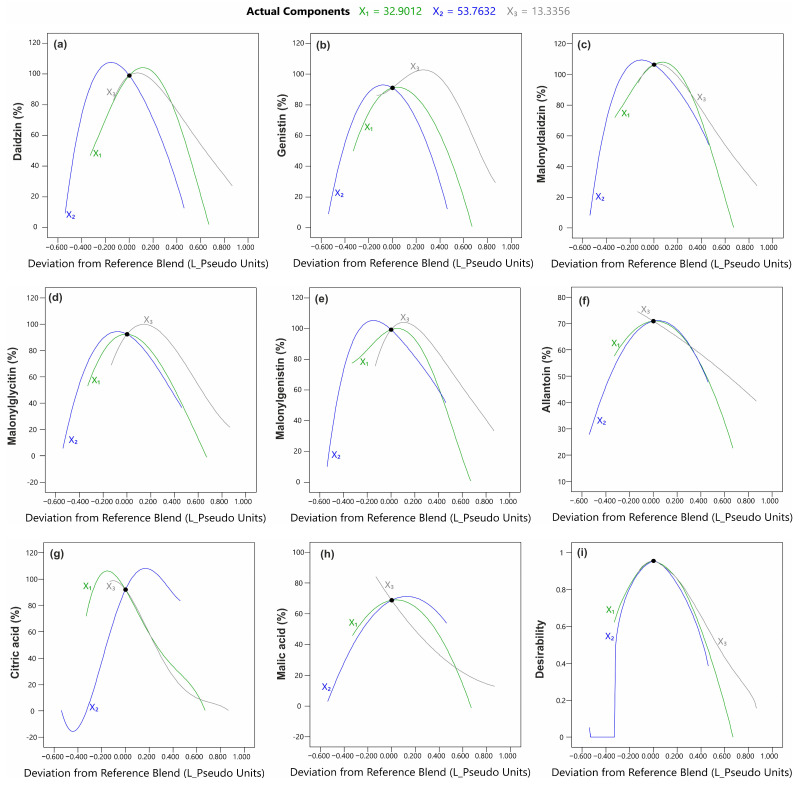
Trace (Piepel) plots for the effect of the solvent composition on the extraction efficiency of (**a**) daidzin, (**b**) genistin, (**c**) 6″-*O*-malonyldaidzin, (**d**) 6″-*O*-malonylglycitin, (**e**) 6″-*O*-malonylgenistin, (**f**) allantoin, (**g**) citric acid, and (**h**) malic acid and (**i**) the overall desirability. Actual components calculated based on numerical optimization: X_1_ (EtOH) = 32.9%; X_2_ (H_2_O) = 53.8%; X_3_ (propanediol) = 13.3% (*v*/*v*/*v*).

To show the impact of the tested solvent composition on each of the evaluated responses, the response surface plots for the effect of the three solvents (EtOH, H_2_O, propanediol) were generated (Figure 2 and Figure 3). Additionally, Piepel trace plots were presented (Figure 4). In the case of isoflavones, the response patterns were mostly similar between the compounds. The shape of the trace plots generally represents a parabolic curve for isoflavones (Figure 4a–e). This proves that the extraction efficiency of isoflavones increased up to approx. 30% with the rise in each solvent amount in the extraction mixture.

It was shown that the three-solvent system provides good efficiency (up to 100% of the total isoflavone content) of the isoflavone extraction. Although the EtOH/H_2_O/propanediol mixture (35:35:30, *v*/*v*/*v*) enabled the extraction of 100% of daidzin, 6″-*O*-malonyldaidzin, and 6″-*O*-malonylgenistin, and over 97% of total 6″-*O*-malonylglycitin and genistin, Yoshiara et al. [16] showed that H_2_O:acetone:EtOH or H_2_O:acetone:acetonitrile are appropriate for the extraction of malonyl-glycosidic and glycosidic isoflavones, respectively. However, the binary mixture of EtOH/H_2_O (50:50, *v*/*v*) for daidzin, genistin, and 6″-*O*-malonyldaidzin, or H_2_O/propanediol (50:50, *v*/*v*) for 6″-*O*-malonylgenistin, provided almost 90% of the total isoflavone extraction yield. In addition, single-composition solvents such as EtOH [40] and methanol [41] were used, and 80% methanol seemed to be the best solvent for phytoestrogen extraction [42]. The obtained results indicated that 99.7% EtOH was not suitable, whereas propanediol provided up to approx. 25% of isoflavone extraction. Furthermore, 100% H_2_O was more appropriate for the extraction of malonyl-glycosides (up to 40–50% of the total extracted compounds) and less appropriate for the extraction of glycoside isoflavones (10% of the total isoflavones).

The phenomenon concerning the achievement of a higher extraction efficiency after the application of binary solutions (alcohol–water mixture) could be a result of the synergistic impact of two extrahents. Water present in the mixture results in swelling and increases the surface contact of the solvent, while alcohol causes the collapse of cells and enhances the infiltration of the mixture [43]. The positive effect of binary water–alcohol solvents has also been observed in the extraction of allantoin or alpha-hydroxy acids. Although the solubility of the allantoin standard is quite weak, below 0.1 g·100 mL^−1^ in the 70% EtOH solution, in comparison with 100% H_2_O (0.66 g·100 mL^−1^) (unpublished data), the binary solvents of water–EtOH or water–propanediol, the latter being a little bit less efficient, exhibited a high ability of allantoin extraction from soybeans, contrary to water used alone or to a mixture of alcohols.

The maximum extraction yield of alpha-hydroxy acids was achieved using approx. 70% H_2_O and 30% alcohol. However, the best solvent mixture composed of EtOH/H_2_O/propanediol was 15:70:15 (*v*/*v*/*v*) for citric acid and 30:70:0 (*v*/*v*/*v*) for malic acid. One of the key factors which determine extraction solvent efficiency is the solubility of the target compound in the solvent mixture [44]. Although some ambiguous solubility results of alpha-hydroxy acids in pure H_2_O and EtOH have been reported [44,45], both acids are generally considered well dissolved in pure EtOH. Surprisingly, the obtained results showed that pure EtOH, propanediol, or a mixture of these two alcohols is not suitable for both citric and malic acid extraction from leaves. It is probable that the same synergistic effect as was mentioned for the binary organic (alcohol) and the water mixture was observed.

The DoE approach gives the opportunity to estimate the optimal parameter setting to obtain a desired response [46]. The numerical optimization using the desirability function has been applied to obtain the optimal mixture composition to provide the maximum extraction effect. Based on the individual desirability of the response of each variable (the extraction effect), the overall desirability was determined at 0.95 (Figure 4i). It is considered that the desirability value between 0.8 and 1.0 is very good and provides an acceptable or excellent product [47]. Here, the optimal concentrations of EtOH, H_2_O, and the propanediol mixture, at the point of the maximum overall desirability, were 32.9%, 53.8%, and 13.3% (*v*/*v*/*v*), respectively. Such a mixture composition enabled the extraction of 99%, 91%, 100%, 92%, 99%, 70%, 92%, and 69% of daidzin, genistin, 6″-*O*-malonyldaidzin, 6″-*O*-malonylglycitin, 6″-*O*-malonylgenistin, allantoin, citric acid, and malic acid, respectively (Figure 4).

### 2.4. Antioxidant Capacity and Soluble Phenol Content

The high ability to scavenge free radicals is a desirable feature of extracts to be used in the preparation of cosmetics [48]. Both ABTS and soluble phenol assays with a Folin–Ciocalteu reagent are considered useful tools for antioxidant property estimation [49].

**Figure 5:**
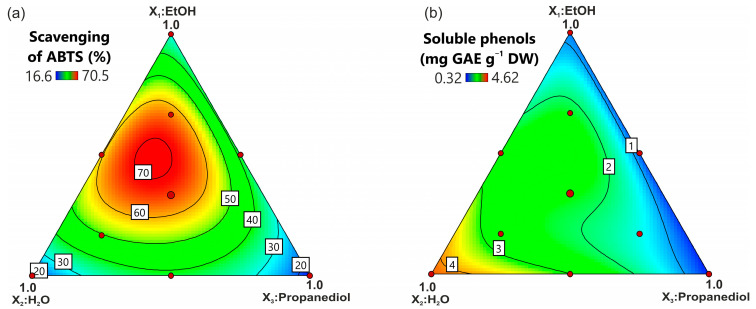
The response contour plot of the impact of the solvent composition on the (**a**) antioxidant capacity and (**b**) soluble phenol content.

The obtained polynomial model for antioxidant activity was highly significant and well fitted (Table S4). It was noted that a three-solvent system of EtOH/H_2_O/propanediol (45:25:20, *v*/*v*/*v*) enabled the maximization of the antioxidant capacity response within the whole tested space (Figure 5). The effect of the solvents on the antioxidant activity of the extracts showed similarities with the isoflavone efficiency pattern (cf. Figure 2). The positive relationship between the antioxidant scavenging ability of the extracts and the amount of extracted isoflavones was proven by the calculated correlation coefficients (Table 3). This phenomenon was expected since isoflavones display free-radical scavenging potential [14]. At the same time, both the allantoin and alpha-hydroxy acids did not exhibit a positive correlation with the scavenging of ABTS (Table 3), which is in accordance with previous reports [18,50]. However, alpha-hydroxy acids are considered antioxidant molecules since they are capable of oxidation inhibition and the suppression of free-radical formation [14], while allantoin exhibits the induction of antioxidant enzyme activity [51]. Interestingly, pure H_2_O turned out to be the most suitable solvent for soluble phenol extraction (Figure 5b). In turn, pure alcohol or a mixture of EtOH or propanediol exhibited a low phenol extraction ability. The obtained results are in accordance with the data presented by Felix et al. [18] who indicated that pure H_2_O extracted 5-fold and 3-fold higher amounts of phenolic compounds from *Fragaria ananassa* compared to pure EtOH or an H_2_O:EtOH (50:50, *v*/*v*) mixture, respectively. Similarly, a high ability of H_2_O for phenolic compound extraction was shown in peppermint [52]. Additionally, the same studies showed that H_2_O:glycerol (70:30, *v*/*v*) or H_2_O:EtOH (50:50, *v*/*v*) mixtures were also efficient in extracting phenolic substances.

### 2.5. Response Prediction and Model Confirmation

The optimal solvent mixture composition developed (32.9% EtOH, 53.9% H_2_O, 13.2% propanediol) has been used to perform the confirmation test (Table 4). Based on the corresponding extracts prepared, the actual experimental values were obtained and compared with predicted values. It was found that both predicted and experimental values corresponded well in all evaluated response variables, with relative deviations between 0.2 and 6.8%. Additionally, no significant differences between the predicted value and the experimental values were detected, except for 6″-*O*-malonyldaidzin with a theoretical value above 100%.

## 3. Materials and Methods

### 3.1. Chemicals and Reference Standard

Chromatographic eluents (acetonitrile, trifluoroacetic acid, sulfuric acid, Merck KGaA, Darmstadt, Germany) and reference standards (daidzein 7-*O*-glucoside, 6″-*O*-malonyldaidzin, genistein 7-*O*-glucoside, 6″-*O*-malonylgenistin, 6″-*O*-malonylglycitin, allantoin, malic acid, citric acid, Merck KGaA, Darmstadt, Germany) were of analytical grade. 1,3-propanediol (99.8% purity) was purchased from Ecospa (Józefosław, Poland), while ethyl alcohol absolute (99.8% purity) was supplied by Avantor Performance Materials Poland S.A. (Gliwice, Poland). Trolox and 2,2′-Azino-bis(3-ethylbenzothiazoline-6-sulfonic acid) diammonium salt (ABTS) were provided by Merck (KGaA, Darmstadt, Germany).

### 3.2. Plant Materials and Extraction Procedure

Soybean seeds (*Glycine max* L.) were purchased from the Enterprise of Horticulture and Nursery in Ożarów Mazowiecki, Poland. After incubation in distilled water (8 h), the seeds were planted into plastic pots filled with garden soil. Plant cultivation was performed in the growth chamber under control conditions (photon flux density of 150 µmol m^−2^ s^−1^, 16/8 h photoperiod, temperature of 24/18 °C, relative humidity of 70%). After 45 days of soil cultivation, the leaves were collected and dried at room temperature for 2 days. Then, the plant material was freeze-dried (0.001 mbar) (Christ Alpha 2–4 LDplus, Martin Christ Gefriertrocknungsanlagen GmbH, Osterode am Harz, Germany) and used for sample preparation. After two-step drying, representative samples of approx. 50 g of leaves were powdered using a laboratory grinder IKA A11 (IKA-Werke, Stufen, Germany) and placed in 2 mL tubes. Then, the samples were subjected to 1 mL of different extraction mixtures (composed of different proportions of EtOH, H_2_O, and propanediol) according to the mixture design plan (see Section 3.5, Table 1) and extracted using an ultrasonic bath (30 min) in the temperature range of 25–38 °C. Afterward, the samples were centrifuged at 10,000× *g* for 5 min and filtered prior to the analysis through a 0.22 µm filter (Chemland, Stargard, Poland). The extraction was repeated using a fresh portion of the mixture until the analytes were exhaustively extracted. The exhaustive extraction was determined as being no signal for the analytes visible on the UHPLC chromatogram. The level of metabolites was monitored using UHPLC.

### 3.3. Secondary Metabolite Analysis

The chromatographic method was applied to determine the phytoestrogens in soybean extracts according to the previous report [2], with minor modifications. Briefly, the ultra-high-performance liquid chromatography (UHPLC) instrument Agilent 1290 Infinity II (Agilent Technologies, Santa Clara, CA, USA), coupled with a diode-array detector (DAD) and an Agilent 6224 electrospray ionization/time-of-flight mass detector (ESI/TOF), was applied for the separation and detection of phytoestrogens in the extracts. The separation was carried out in RP18 Titan reversed-phase column (Supelco, Sigma-Aldrich, Burlington, MA, USA) (10 cm × 2.1 mm i.d., 1.9 µm particle size) using a mixture of water with 0.05% formic acid (solvent A) and acetonitrile with 0.05% formic acid (solvent B). The gradient program was as follows: 0–5 min A 95%, B 5%; 5–15 min A 95–85%, B 5–15%; 15–40 min A 85–75%, B 15–25%; 40–45 min A 75%, B 25%. The UV-VIS spectral data were collected in a wavelength range from 190 to 400 nm; however, the quantification of phytoestrogens was performed at 256 nm. The mass spectrometry analysis was carried out with the following parameters: gas temperature of 325 °C, gas flow of 5 L^−1^, nebulizer pressure of 30 psi, capillary voltage of 3500 V, fragmentator—200 V, skimmer—65 V, and ion acquisition range of 100–1050 *m*/*z* with a scan rate of 1.00 (spectra·s^−1^).

Both the allantoin and carboxylic acids were analyzed using high-performance liquid chromatography (HPLC) coupled with DAD (VWR Hitachi Chrmoaster 600 Merck, Darmstadt, Germany) and a Razex ROA-Organic H+ (8%) LC column (300 × 7.8 mm) (Phenomenex Inc., Torrance, CA, USA). Isocratic elution using water with 0.0025 M H_2_SO_4_ was applied for the separation of metabolites. Allantoin was recorded at 195 nm, and malic and citric acids were recorded at 210 nm.

The compounds were identified based on the comparison of UV-VIS and MS spectrum data and retention times with reference standards. All compounds were quantified according to the calibration curves of reference standards. The metabolite content was calculated as a percentage of the total amount of the compound in the plant materials.

### 3.4. Antioxidant Properties and Soluble Phenol Assay

The antioxidant capacity was determined using ABTS and expressed as milligrams of Trolox equivalent per gram of DW [53]. The total soluble phenols were estimated using the Folin–Ciocalteau reagent and calculated as gallic acid equivalent per gram of dry material [54].

### 3.5. Experimental Design and Optimization of Solvent Composition

**Figure 6:**
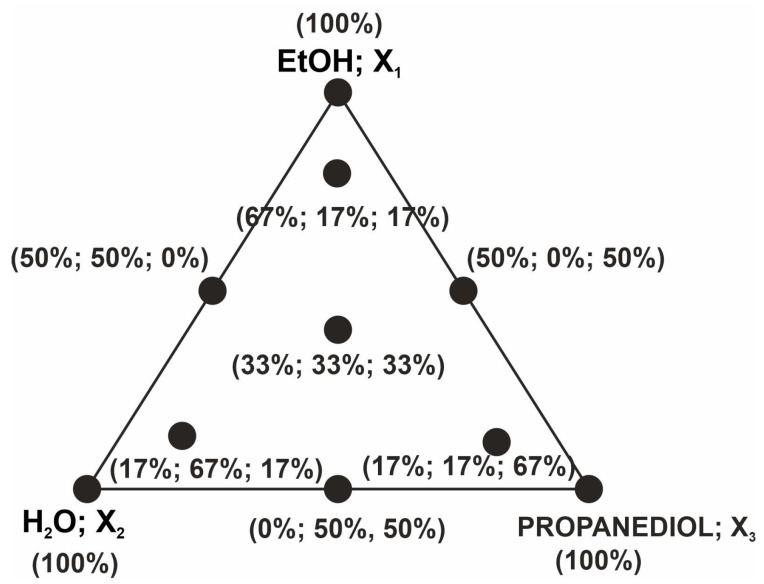
Experimental point distributions.

Using a simplex centroid design augmented with interior points, 10 different solvent compositions of EtOH (X_1_), H_2_O (X_2_), and propanediol (X_3_) were selected (Table 2, Figure 6). The whole experiment was repeated 3 times.

Based on the chromatographic analysis of each compound extracted using selected solvent mixtures, polynomial models were developed to represent the response (the amount of the extracted compound) in relation to solvent composition. The models developed, as well as their components and the lack-of-fit models, were verified using ANOVA at *p* < 0.05 with a null hypothesis (not significant), the lack of correlations between the variable and the response. Additionally, the normal distribution of residuals was checked using the Shapiro–Wilk test (*p* < 0.05). The adequacy of the models was estimated using the coefficient of determination (*R*^2^), adjusted *R*^2^, predicted *R*^2^, and adequate precision expressed as a signal-to-noise ratio. The obtained polynomial models were used to determine overall desirability [46] and to establish an optimal solvent mixture composition, which allowed the maximization of the extraction yield response. The differences between the predicted value and the experimental values were evaluated using a one-sample *t*-test. Statistical analyses were performed using both Statistica ver. 13.3.0.3 (Tibco Software Inc., Palo Alto, CA, USA) and Design Expert ver. 13 (Stat-Ease Inc., Minneapolis, MN, USA).

## 4. Conclusions

The utilization of a simplex centroid mixture design in the optimization of metabolite extraction from soybean leaves turned out to be a useful cost-effective method. Despite the different chemical nature of targeted compounds, a three-solvent system composed of EtOH:H_2_O:propanediol enabled the extraction of isoflavones (daidzin, genistin, 6″-*O*-malonyldaidzin, 6″-*O*-malonylglycitin, 6″-*O*-malonylgenistin), allantoin, and alpha-hydroxy acids. The polynomial models developed were excellently fitted with good prediction ability. Interestingly, the models developed for all isoflavones showed the lowest EtOH coefficients among all the solvents, but this solvent exhibited a high interaction with other tested extrahents, especially with water. The maximum overall desirability was achieved using a mixture consisting of 32.9% EtOH, 53.8% H_2_O, and 13.3% propanediol (*v*/*v*/*v*). Such a mixture provided a good extraction efficiency of the metabolites from soybean leaves and a good level of free-radical scavenging. The applied confirmation model procedure showed that the experimental and predicted values corresponded well with each other. The procedure allowed for limiting additional extraction steps, such as evaporation of toxic solvents and thus the results of extraction are suitable for direct use in medical products and cosmetic preparations.

## Figures and Tables

**Table 1:** The total content of isoflavones, allantoin, and alpha-hydroxy acids in soybean leaves.

Isoflavones (mg·g^−1^ DW)		Allantoin (mg·g^−1^ DW)	
**Daidzin**			
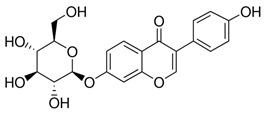	0.310	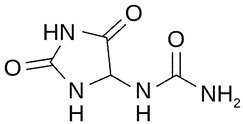	5.31
**Genistin**			
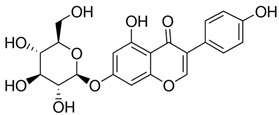	0.358		
		**Alpha-hydroxy acids (mg·g^−1^ DW)**	
**6″-*O*-malonyldaidzin**		**Malic acid**	
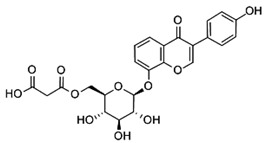	0.719	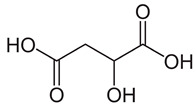	13.38
**6″-*O*-malonylglycitin**		**Citric acid**	
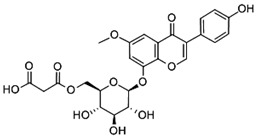	0.179	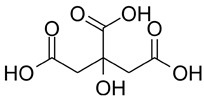	7.98
**6″-*O*-malonylgenistin**			
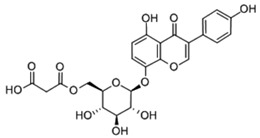	0.840		

**Table 2:** Experimental factors and the measured values of responses. Variable code: X_1_—EtOH, X_2_—H_2_O, X_3_—propanediol. The response was measured in triplicate ± SD.

Independent Variables			Responses									
EtOH X_1_ (%)	H_2_O X_2_ (%)	Propanediol X_3_ (%)	Daidzin (%)	Genistin (%)	6″-*O*-Malonyldaidzin (%)	6″-*O*-Malonylglycitin (%)	6″-*O*-Malonylgenistin (%)	Allantoin (%)	Malic Acid (%)	Citric Acid (%)	ABTS Scavenging (%)	Soluble Phenols (mg GAE g^−1^)
100	0	0	0.9 ± 0.43	0.3 ± 0.37	nd	nd	nd	19.9 ± 2.73	0.8 ± 0.09	nd	31.7 ± 0.42	1.07 ± 0.24
0	100	0	12.6 ± 1.35	11.8 ± 1.09	54.9 ± 1.91	37.2 ± 3.14	51.5 ± 3.45	48.3 ± 3.23	51.6 ± 1.89	83.5 ± 2.12	17.9 ± 0.03	4.28 ± 0.48
0	0	100	26.1 ± 4.32	27.7 ± 3.35	28.5 ± 2.42	22.2 ± 3.11	32.8 ± 2.09	37.2 ± 1.12	6.8 ± 0.76	nd	16.6 ± 0.74	0.62 ± 0.23
50	50	0	85.8 ± 0.62	90.5 ± 1.22	92.8 ± 1.50	67.9 ± 4.24	71.3 ± 4.21	76.4 ± 0.96	82.2 ± 5.24	91.0 ± 1.41	59.4 ± 0.91	2.34 ± 0.12
0	50	50	68.8 ± 2.49	74.2 ± 4.83	79.4 ± 4.22	60.1 ± 5.12	88.2 ± 5.26	66.5 ± 3.97	39.9 ± 2.43	49.4 ± 0.81	37.8 ± 3.18	2.99 ± 0.44
50	0	50	14.7 ± 4.90	16.1 ± 2.52	12.5 ± 2.30	8.9 ± 2.35	14.4 ± 2.45	37.6 ± 2.98	7.5 ± 0.39	1.1 ± 0.31	37.5 ± 2.97	0.33 ± 0.02
33.3	33.3	33.3	96.1 ± 1.01	94.8 ± 2.04	99.1 ± 1.04	98.1 ± 1.33	95.6 ± 4.06	56.6 ± 3.21	53.4 ± 2.50	32.7 ± 0.89	68.3 ± 2.40	2.09 ± 0.39
66.7	16.7	16.7	79.9 ± 0.86	52.2 ± 5.30	76.3 ± 4.04	53.8 ± 2.55	79.5 ± 3.26	56.2 ± 4.12	23.8 ± 0,98	nd	60.9 ± 1.34	2.33 ± 0.33
16.7	66.7	16.7	78.2 ± 5.29	81.3 ± 5.56	89.1 ± 5.37	81.6 ± 4.63	91.9 ± 0.30	64.8 ± 1.79	54.7 ± 3.65	99.3 ± 5.65	48.9 ± 1.48	2.80 ± 0.35
16.7	16.7	66.7	60.9 ± 4.47	82.2 ± 1.13	56.4 ± 4.45	58.3 ± 3.75	69.9 ± 6.15	52.4 ± 5.03	23.7 ± 2.71	3.9 ± 0.05	42.0 ± 5.42	1.52 ± 0.21

nd—not detected.

**Table 3:** Pearson’s correlation coefficients for the antioxidant capacity, soluble phenols, and metabolite concentrations in the extracted solutions.

	Daidzin	Genistin	6″-*O*-Malonyldaidzin	6″-*O*-Malonylglycitin	6″-*O*-Malonylgenistin	Allantoin	Citric Acid	Malic Acid
ABTS scavenging	0.81 ***	0.77 ***	0.67 **	0.71 ***	0.58 **	ns	ns	ns
Soluble phenols	ns	ns	0.61 **	0.51 *	0.58 **	0.64 *	0.76 ***	0.69 *

ns—non-significant; * *p* < 0.05; ** *p* < 0.01; *** *p* < 0.001.

**Table 4:** Predicted and mean (±SD) experimental values at the optimal extraction conditions of 32.9% ethanol (X_1_), 53.9% water (X_2_), and 13.2% propanediol.

Response Variables	Predicted Value	Experimental Value	RD (%)
Daidzin (%)	98.9	97.7 ± 2.31	−1.21
Genistin (%)	90.9	90.7 ± 2.56	−0.22
6″-*O*-Malonyldaidzin (%)	106.3	99.1 ± 0.99	−6.77
6″-*O*-Malonylglycitin (%)	92.2	90.8 ± 3.11	−1.52
6″-*O*-Malonylgenistin (%)	99.1	99.3 ± 1.26	0.20
Allantoin (%)	70.9	70.1 ± 1.30	−1.13
Citric acid (%)	92.3	91.2 ± 1.73	−1.19
Malic acid (%)	68.9	68.3 ± 0.71	−0.87
ABTS (mg TE L^−1^)	63.2	60.6 ± 2.21	−4.11
Soluble phenols (mg GAE L^−1^)	2.52	2.30 ± 0.16	−8.73

RD—response deviation; ABTS—2,2′-Azino-bis(3-ethylbenzothiazoline-6-sulfonic acid) diammonium salt; TE—Trolox equivalent; GAE—gallic acid equivalent.

## Data Availability

The data presented in this study are available on request from the corresponding author.

## Supplementary Materials

The following supporting information can be downloaded from https://www.mdpi.com/article/10.3390/molecules28093963/s1, Figure S1: The mass spectrometer scans of (a) daidzin, (b) genistin, (c) 6″-O-malonyldaidzin, (d) 6″-O-malonylglycitin, and (e) 6″-O-malonylgenistin extracted from the soybean leaves; Figure S2: A representative HPLC-DAD chromatogram (210 nm) of the soybean leaf extract: (1) citric acid; (2) malic acid; (3) allantoin; Table S1: A summary of different experimental conditions for isoflavone extraction from soybean; Table S2: The mass spectrometry data of the components identified in soybean leaves in the positive ionization mode; Table S3: Fit statistics, ANOVA, and regression coefficients of the models for five isoflavones, allantoin, and alpha-hydroxy acids extracted using a simplex centroid mixture design with three solvents, i.e., EtOH—X1, H2O—X2, and propanediol—X3. The significance level was set at α = 0.05; Table S4: Fit statistics, ANOVA, and regression coefficients of the models of the antioxidant capacity and soluble phenols, using a simplex centroid mixture design with three solvents, i.e., EtOH—X_1_, H_2_O—X_2_, and propanediol—X_3_. The significance level was set at α = 0.05. Refs. [55,56,57,58,59] are cited in the Supplementary Materials.

## Sample Availability

Samples of the compounds are available from the authors.
