# Supplementary material for: Extraction of Isoflavones, Alpha-Hydroxy Acids, and Allantoin from Soybean Leaves—Optimization by a Mixture Design of the Experimental Method
Source: Molecules. 2023 May 8;28(9):3963. doi: 10.3390/molecules28093963 (PMC10179801; doi:10.3390/molecules28093963)
Supplement: Supplementary file 1 [file molecules-28-03963-s001.zip › molecules-2354892-supplementary.pdf]

molecules

MDPI

SUPPLEMENTARY MATERIALS

Extraction of Isoflavones, Alpha-Hydroxy Acids, and Allantoin from Soybean Leaves—Optimization by a Mixture Design of the Experimental Method

Sławomir Dresler [1,2,*], Maciej Strzemski [1], Izabela Baczewska [1], Mateusz Koselski [2], Mohammad Bagher Hassanpouraghdam [3], Dariusz Szczepanek [4], Ireneusz Sowa [1], Magdalena Wójciak [1] and Agnieszka Hanaka [2]

[1] Department of Analytical Chemistry, Medical University of Lublin, Chodźki 4a, 20-093 Lublin, Poland; maciejstrzemski@umlub.pl (M.S.); i.sowa@umlub.pl (I.S.); magdalenawojciak@umlub.pl (M.W.)
[2] Department of Plant Physiology and Biophysics, Institute of Biological Sciences, Maria Curie-Skłodowska University, Akademicka 19, 20-033 Lublin, Poland; agnieszka.hanaka@mail.umcs.pl (A.H.)
[3] Department of Horticulture, Faculty of Agriculture, University of Maragheh, Maragheh 5518183111, Iran
[4] Chair and Department of Neurosurgery and Paediatric Neurosurgery, Medical University of Lublin, 20-090 Lublin, Poland
* Correspondence: slawomir.dresler@umlub.pl

Citation: Dresler, S.; Strzemski, M.; Baczewska, I.; Koselski, M.; Hassanpouraghdam, M.B.; Szczepanek, D.; Sowa, I.; Wójciak, M.; Hanaka, A. Extraction of Isoflavones, Alpha-Hydroxy Acids, and Allantoin from Soybean Leaves—Optimization by a Mixture Design of the Experimental Method. *Molecules* **2023**, *28*, 3963. https://doi.org/10.3390/molecules28093963

Academic Editor: Elena Falqué López

Received: 3 April 2023
Revised: 27 April 2023
Accepted: 4 May 2023
Published: 8 May 2023

Copyright: © 2023 by the authors. Licensee MDPI, Basel, Switzerland. This article is an open access article distributed under the terms and conditions of the Creative Commons Attribution (CC BY) license (https://creativecommons.org/licenses/by/4.0/).

Table S1. Summary of different experimental conditions for isoflavones extraction from soybean.

Plant material	Experimental conditions	Best conditions based on isoflavones yield extraction	References
Soy flour	a mixture of four solvents: water, acetone, ethanol, acetonitrile	water:acetone:ethanol (2:1:1) for total forms and malonyl-glycosidic forms; water:acetone:acetonitrile (2:1:1) for glycosidic isoflavones; water:acetone (1:1) for aglycone	[16]
Soy flour	ethanol concentration 60-100%; temperature 40-70°C; extraction time 45-90 min; solid/liquid ratio 1/15-1/30	72.5°C; 67.5 min extraction time; 1/26.5 solid/liquid ratio; 80% (v/v) ethanol	[55]
Soy flour	ethanol concentration 40-100%; temperature 40-70°C; extraction time 30-80 min; solid/liquid ratio 1/1 – 1/25; pH 7-12	70°C; pH 9.0; 60 min extraction time; 1/15 solid/liquid ratio; 65% (v/v) ethanol; 94.34% extraction yield of isoflavones	[56]
Soy flour	ethanol concentration 50-100%; temperature 25-85°C; extraction time 30-180 min	44°C; 105 min extraction time; 78% (v/v) ethanol	[57]
Soybean umbilicus	ethanol concentration 40-90%; temperature 40-80°C; solid/liquid ratio 1/10 – 1/35; extraction time 30-180 min	80°C; 90 min extraction time; 1/35 solid/liquid ratio; 80% (v/v) ethanol; 90% extraction yield of isoflavones	[58]
Soybean seeds	ethanol concentration 50-90%; solvent/solid ratio 10-20 mL g^{-1}; 30-70°C	60°C; 70% (v/v) ethanol; solvent/solid ratio 20 mL g^{-1}	[59]

Table S2. The mass spectrometry data of the components identified in soybean leaves in the positive ionization mode.

TR (min)	Observed Ion Mass $[M-H]^+$	Δ ppm	Formula	Identified
21.52	417.11905 (255)	2.5	C21H20O9	Daidzin
28.64	433.11299 (271)	0.15	C21H20O10	Genistin
33.52	503.11881 (255)	0.81	C24H22O12	6″-*O*-Malonyldaidzin
35.67	533.12981 (285)	1.58	C25H24O13	6″-*O*-Malonylglycitin
41.27	519.11444 (271)	2.17	C24H22O13	6″-*O*-Malonylgenistin

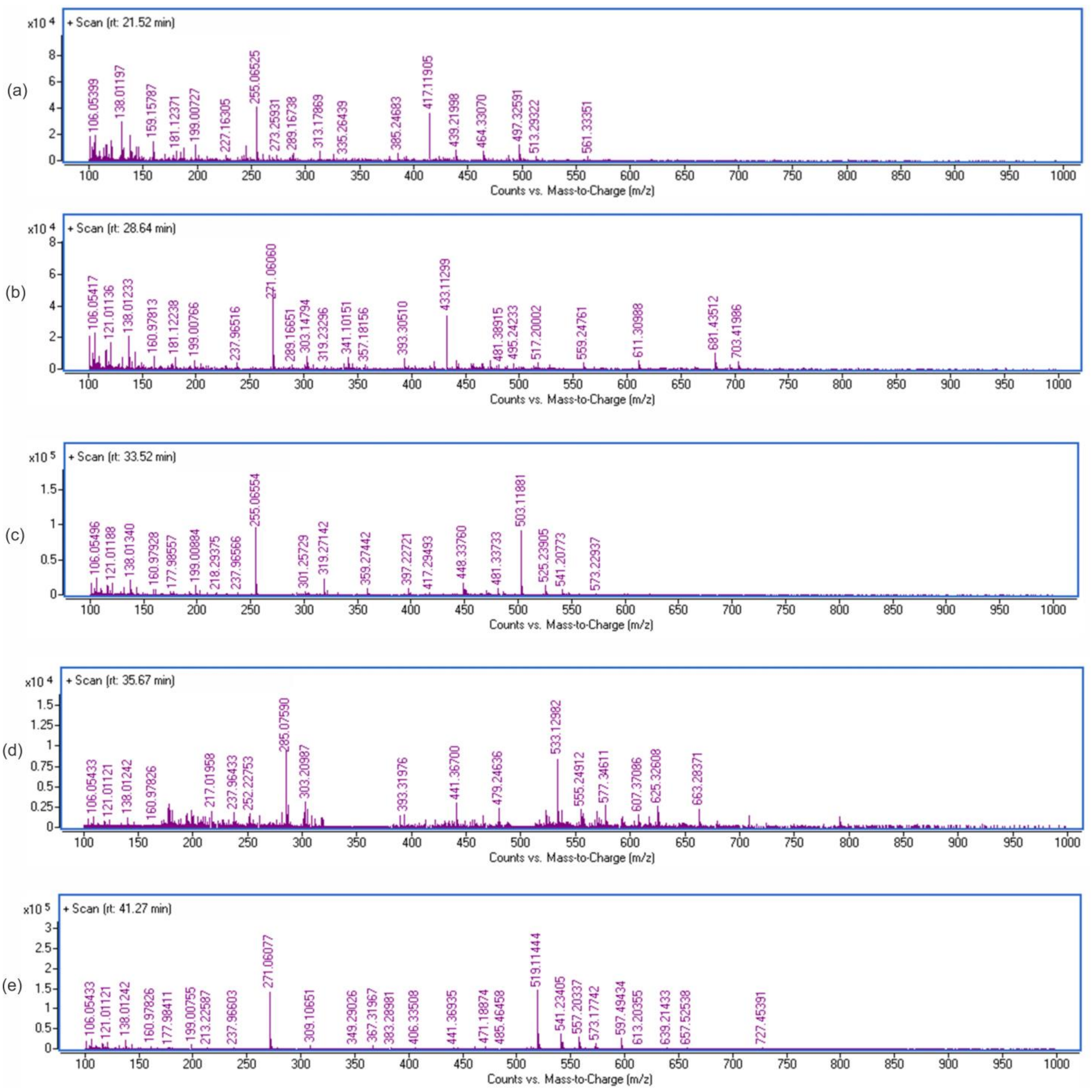

Figure S1. The mass spectrometer scans of (a) daidzin; (b) genistin; (c) 6″-*O*-malonyldaidzin; (d) 6″-*O*-malonylglycitin; (e) 6″-*O*-malonylgenistin extracted from soybean leaves.

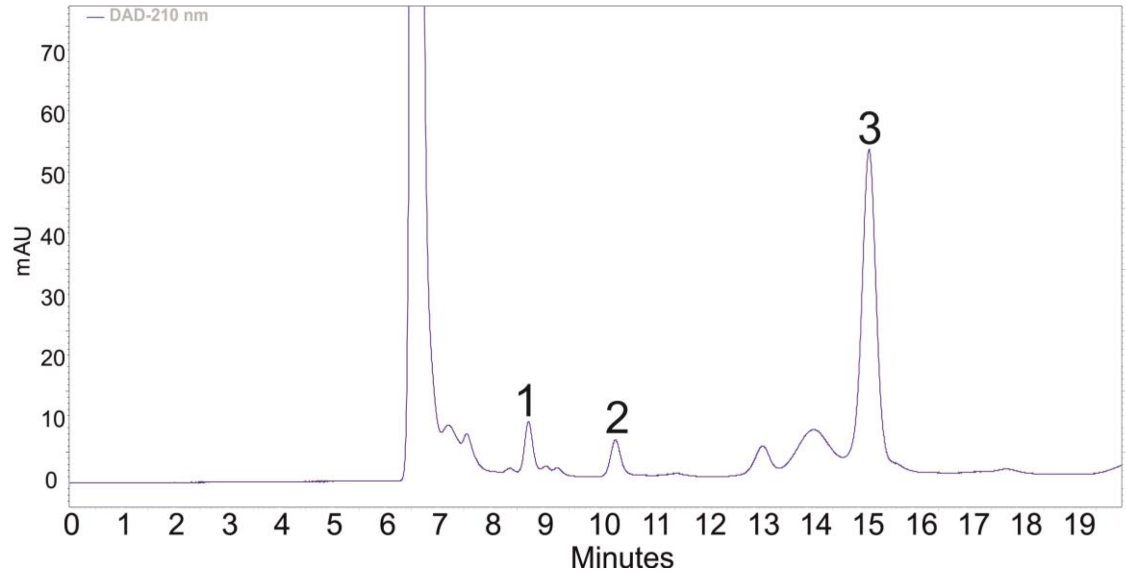

Figure S2. A representative HPLC-DAD chromatogram (210 nm) of the soybean leaves extract, (1) citric acid; (2) malic acid; (3) allantoin.

Table S3. Fit statistics, ANOVA, and regression coefficients of the models for five isoflavones, allantoin, and alpha-hydroxy acids extracted using a simplex centroid mixture design with three solvents, i.e., EtOH – X_1, H_2O – X_2, and propanediol – X_3. The significance level was set at α = 0.05.

**Daidzin**	R^2 0.9814		**Adj** R^2 0.9748	**Pred** R^2 0.9703	**Adeq Precision** 32.5024	
			**ANOVA**			
**Component**	**Coefficient**	**Std. Error**	**Source**	**Sum of Squares**	***F*-value**	***p*-value**
			**Model**	22232.50	147.89	<0.0001
X_1	1.97	3.46	**Linear Mixture**	1083.11	18.01	0.0001
X_2	12.58	3.77				
X_3	27.11	3.34				
X_1X_2	315.38	18.38		8849.71	294.34	<0.0001
X_2X_3	196.85	17.93		3625.26	120.57	<0.0001
$(X_1)^2X_2X_3$	2331.51	289.16		1954.69	65.01	<0.0001
			**Residual**	420.93		
			**Lack of Fit**	58.07	0.4000	0.8044
			**Pure Error**	362.87		
			**Cor Total**	22653.44		
**Genistin**	R^2 0.9898		**Adj** R^2 0.9855	**Pred** R^2 0.9789	**Adeq Precision** 37.8537	
			**ANOVA**			
**Component**	**Coefficient**	**Std. Error**	**Source**	**Sum of Squares**	***F*-value**	***p*-value**
			**Model**	23393.30	232.57	<0.0001
X_1	0.6867	2.89	**Linear Mixture**	2274.76	56.54	<0.0001
X_2	12.12	3.10				
X_3	29.37	2.83				
X_1X_2	333.14	14.84		10136.23	503.87	<0.0001
X_1X_3	215.71	15.04		4138,14	205.70	<0.0001
$(X_1X_2X_3)^2$	1916.77	236.53		1321.14	65.67	<0.0001
			**Residual**	241.40		
			**Lack of Fit**	70.88	1.25	0.3491
			**Pure Error**	170.52		
			**Cor Total**	23634.71		
6″-*O*-**Malonyldaidzin**	R^2 0.9869		**Adj** R^2 0.9819	**Pred** R^2 0.9780	**Adeq Precision** 41.1449	
			**ANOVA**			
**Component**	**Coefficient**	**Std. Error**	**Source**	**Sum of Squares**	***F*-value**	***p*-value**
			**Model**	18219.99	196.29	<0.0001
X_1	0.3107	3.50	**Linear Mixture**	5160.35	138.99	<0.0001
X_2	54.20	2.96				
X_3	27.53	2.66				
X_1X_2	257.57	15.07		5424.96	292.22	<0.0001
X_2X_3	145.07	14.17		1946.37	104.84	<0.0001
$(X_1)^2X_2X_3$	2255.92	232.50		1747.73	94.14	<0.0001
			**Residual**	241.34		
			**Lack of Fit**	55.35	0.6696	0.6292
			**Pure Error**	185.99		
			**Cor Total**	18461.32		
6″-*O*-**Malonylglycitin**	R^2 0.9883		**Adj** R^2 0.9834	**Pred** R^2 0.9657	**Adeq Precision** 40.2689	
			**ANOVA**			
**Component**	**Coefficient**	**Std. Error**	**Source**	**Sum of Squares**	***F*-value**	***p*-value**
			**Model**	17634.78	202.00	<0.0001
X_1	–0.9398	2.56	**Linear Mixture**	4458.81	127.68	<0.0001

X_2	36.57	2.86				
X_3	21.66	2.69				
X_1X_2	197.04	14.20		3359.91	192.43	<0.0001
X_1X_3	122.26	14.19		1295.64	74.20	<0.0001
$X_1X_2X_3$	1124.13	89.13		2777.38	159.07	<0.0001
			**Residual**	209.53		
			**Lack of Fit**	32.03	19.72	0.6660
			**Pure Error**	177.50		
			**Cor Total**	17844.31		

**6″-*O*-Malonylgenistin**	R^2 0.9681		**Adj** R^2 0.95567	**Pred** R^2 0.9371	**Adeq Precision** 27.1575	
			**ANOVA**			
**Component**	**Coefficient**	**Std. Error**	**Source**	**Sum of Squares**	***F*-value**	***p*-value**
			**Model**	20083.41	84.90	<0.0001
X_1	0.8029	4.34	**Linear Mixture**	6074.06	64.19	<0.0001
X_2	51.74	4.73				
X_3	33.33	4.18				
X_1X_2	183.21	23.06		2986.49	63.12	<0.0001
X_1X_3	184.92	22.49		3199.28	67.62	<0.0001
$(X_1)^2X_2X_3$	2720.76	362.73		2661.84	56.26	<0.0001
			**Residual**	662.37		
			**Lack of Fit**	257.65	1.59	0.2508
			**Pure Error**	404.72		
			**Cor Total**	20745.78		

**Allantoin**	R^2 0.9240		**Adj** R^2 0.8805	**Pred** R^2 0.7796	**Adeq Precision** 12.8656	
			**ANOVA**			
**Component**	**Coefficient**	**Std. Error**	**Source**	**Sum of Squares**	***F*-value**	***p*-value**
			**Model**	3227.06	21.27	0.0005
X_1	22.65	4.01	**Linear Mixture**	1500.15	19.77	0.0013
X_2	47.66	5.90				
X_3	40.55	5.23				
X_1X_2	154.58	94.98		1426.95	37.61	0.0005
X_2X_3	71.18	26.78		268.07	7.07	0.0326
			**Residual**	265.57		
			**Lack of Fit**	210.71	1.54	0.4393
			**Pure Error**	54.86		
			**Cor Total**	3492.62		

**Citric acid**	R^2 0.9996		**Adj** R^2 0.9993	**Pred** R^2 0.9985	**Adeq Precision** 141.6698	
			**ANOVA**			
**Component**	**Coefficient**	**Std. Error**	**Source**	**Sum of Squares**	***F*-value**	***p*-value**
			**Model**	22356.21	3440.24	<0.0001
X_1	0.3333	0.6219	**Linear Mixture**	17810.89	9592.79	<0.0001
X_2	83.50	0.6813				
X_3	0.3333	0.6219				
X_1X_2	196.33	14.84		3304.01	3559.02	<0.0001
X_2X_3	30.05	15.04		77.40	83.37	<0.0001
$(X_1)^2X_2X_3$	−3424.90	236.53		1524.46	1642.12	<0.0001
$X_1(X_2)^2X_3$	3262.01	143.44		480.10	517.15	<0.0001
$(X_1X_2X_3)^2$	−1499.87	84.52		292.35	314.91	<0.0001
			**Residual**	9.28		
			**Lack of Fit**	1.33	1.51	0.2504

			**Pure Error**	7.95		
			**Cor Total**	23634.71		
**Malic acid**	R^2 0.9429		**Adj** R^2 0.9184	**Pred** R^2 0.8759	**Adeq Precision** 18.5481	
			**ANOVA**			
**Component**	**Coefficient**	**Std. Error**	**Source**	**Sum of Squares**	F**-value**	p**-value**
			**Model**	6178.50	38.50	<0.0001
X_1	−1.08	6.27	**Linear Mixture**	3679.65	34.39	0.0002
X_2	53.85	6.27				
X_3	12.90	5.36				
X_1X_2	217.36	31.80		2498.85	46.71	0.0002
			**Residual**	374.47		
			**Lack of Fit**	368.20	9.77	0.2401
			**Pure Error**	6.28		
			**Cor Total**	6552.98		

Table S4. Fit statistics, ANOVA and regression coefficients of the models of the antioxidant capacity and soluble phenols, using a simplex centroid mixture design with three solvents, i.e., EtOH – X_1, H_2O – X_2, and propanediol – X_3. The significance level was set at $\alpha = 0.05$.

**Scavenging of ABTS**	R^2 0.9896		**Adj** R^2 0.9839	**Pred** R^2 0.9738	**Adeq Precision** 34.3919	
			**ANOVA**			
**Component**	**Coefficient**	**Std. Error**	**Source**	**Sum of Squares**	***F*-value**	***p*-value**
			**Model**	5510.33	174.32	<0.0001
			**Linear Mixture**	919.68	87.28	<0.0001
X_1	30.95	1.58				
X_2	17.85	1.59				
X_3	16.57	1.62				
X_1X_2	139.12	7.79		1682.01	319.26	<0.0001
X_1X_3	55.96	7.90		264.27	50.16	<0.0001
X_2X_3	84.65	7.67		640.92	121.65	<0.0001
$(X_1)^2X_2X_3$	1047.16	130.86		337.39	64.04	<0.0001
			**Residual**	57.95		
			**Lack of Fit**	26.34	13.17	0.0654
			**Pure Error**	31.62		
			**Cor Total**	5568.29		

**Soluble phenols**	R^2 0.9384		**Adj** R^2 0.9164	**Pred** R^2 0.8626	**Adeq Precision** 20.0275	
			**ANOVA**			
**Component**	**Coefficient**	**Std. Error**	**Source**	**Sum of Squares**	***F*-value**	***p*-value**
			**Model**	25.13	42.64	<0.0001
			**Linear Mixture**	22.78	96.67	<0.0001
X_1	0.8623	0.2028				
X_2	4.23	0.2173				
X_3	0.4607	0.2083				
X_2X_3	2.66	1.15		0.6342	5.38	0.0360
$(X_1)^2X_2X_3$	81.98	22.22		1.60	13.61	0.0024
$(X_1X_2)^2X_3$	-72.98	22.78		1.21	10.26	0.0064
			**Residual**	1.65		
			**Lack of Fit**	0.5537	1.26	0.3467
			**Pure Error**	1.10		
			**Cor Total**	26.78		

Disclaimer/Publisher's Note: The statements, opinions and data contained in all publications are solely those of the individual author(s) and contributor(s) and not of MDPI and/or the editor(s). MDPI and/or the editor(s) disclaim responsibility for any injury to people or property resulting from any ideas, methods, instructions or products referred to in the content.
